# Microbial Spy Games and Host Response: Roles of a *Pseudomonas aeruginosa* Small Molecule in Communication with Other Species

**DOI:** 10.1371/journal.ppat.1002312

**Published:** 2011-11-17

**Authors:** Lucja M. Jarosz, Ekaterina S. Ovchinnikova, Michael M. Meijler, Bastiaan P. Krom

**Affiliations:** 1 Department of Biomedical Engineering, W.J. Kolff Institute, University Medical Center Groningen and the University of Groningen, Groningen, The Netherlands; 2 Department of Chemistry and National Institute for Biotechnology in the Negev, Ben-Gurion University of the Negev, Be'er Sheva, Israel; Duke University Medical Center, United States of America

## Introduction

Gathering and sharing of information is extremely important in human society. Especially in times of war, the difference between victory and defeat can depend on the ability to obtain, encrypt, and share information, and sophisticated systems have been developed for exactly this purpose. Similarly, in their constant battles with competitors and the host immune system, (opportunistic) microbial pathogens have developed sophisticated cell–cell communication systems termed quorum sensing (QS) that allow exchange of critical information. In return, competing microbes, as well as the host immune system, have developed means to intercept and decode these messages. The information obtained by this molecular espionage is used for their benefit, either to win the war (microbe against microbe), or to prepare for an upcoming battle (microbe against immune system). To illustrate the clinical importance of this microbial spy game, we will focus on the biological activity of a single bacterial QS molecule on surrounding microbes and the host immune system and its diverse “meaning” to different receivers. Infections related to burn wounds, cystic fibrosis, and periodontal diseases consist most commonly of the bacteria *Pseudomonas aeruginosa* and *Staphylococcus aureus* and the fungus *Candida albicans,* and represent niches with an active host response. Therefore, we will specifically provide five facts about how the *P. aeruginosa* QS molecule 3-oxo-dodecanoyl-L-homoserine lactone (3OC_12_HSL) plays a pivotal role in this triangle of interspecies interactions and how microbial behavior elicited by 3OC_12_HSL has consequences on host response.

## Quorum Sensing: A Sophisticated Communication System

QS is a system that enables microbes to monitor population cell density through the production, secretion, and sensing of small diffusible molecules [1]. When such molecules reach a threshold concentration, microbial cells in the vicinity detect the signal and coordinately respond by modifying their gene expression; often these genes are associated with virulence and pathogenesis. Several different types of QS molecules have been described for a wide variety of microbial species.

**Figure 1:**
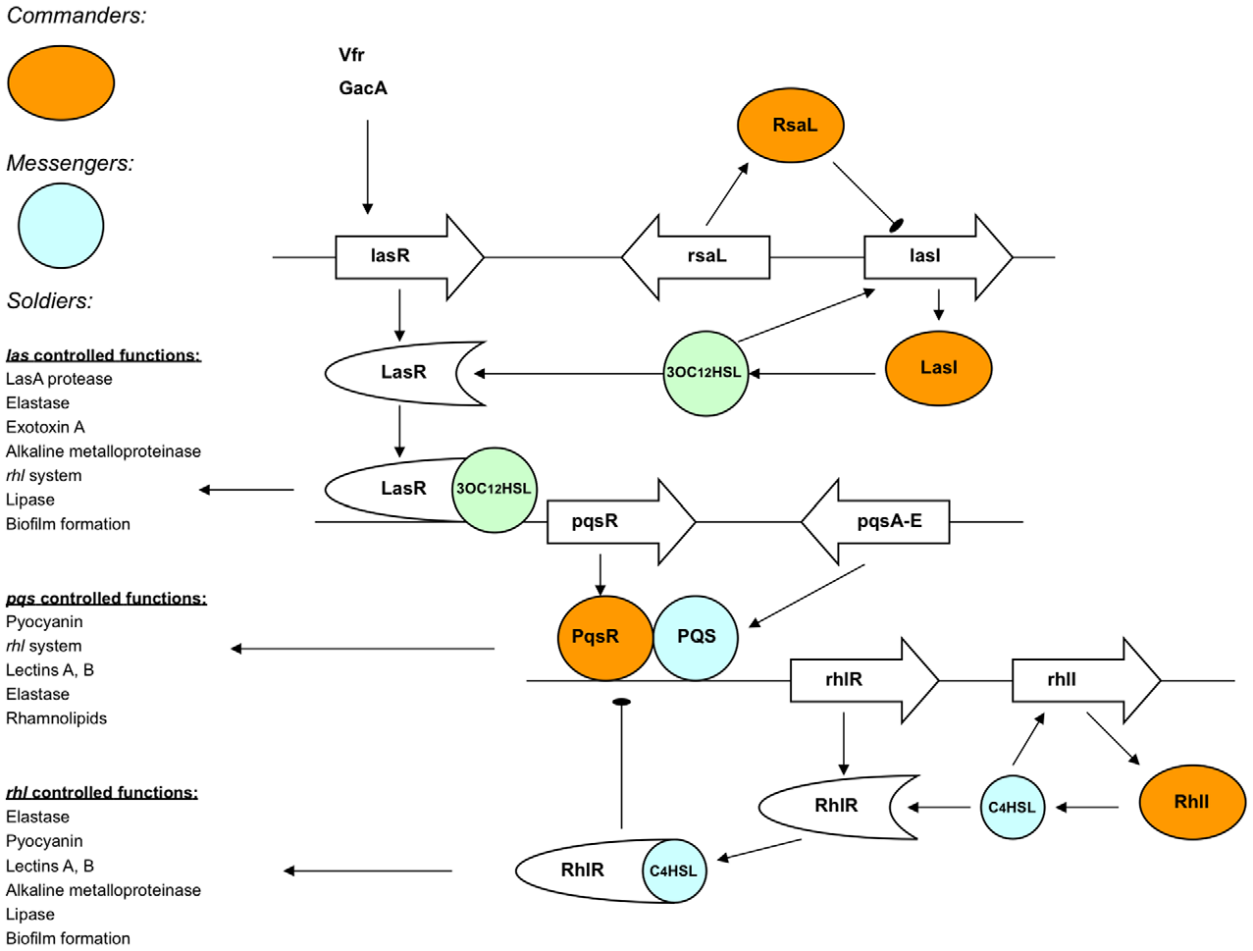
Schematic overview of the complex QS systems present in *P. aeruginosa*. The commanders represent the major regulatory systems, while the messengers are the signaling molecules. The soldiers represent the various virulence factors that have a function in the interaction of *P. aeruginosa* with the host.

In the Gram-negative pathogenic bacterium *P. aeruginosa*, the QS system is perhaps the most complex because several distinct QS sub-systems are hierarchically intertwined at different stages [2] (Figure 1). The best-studied of these systems is the LasI/R, which consists of the LasI protein that catalyzes the synthesis of the diffusible molecule 3OC_12_HSL [3]. Intracellular accumulation of 3OC_12_HSL is sensed by the receptor LasR and induces expression of several virulence factors, such as exotoxins and proteases, and production of secondary metabolites. Significantly, LasR is also responsible for the development and maturation of biofilms, which are communities of surface-adherent microbial species implicated in chronic and resistant infections such as burn wounds, pneumonia in cystic fibrosis patients, and periodontitis [4].

## Host Immune Recognition of 3OC_12_HSL

**Figure 2:**
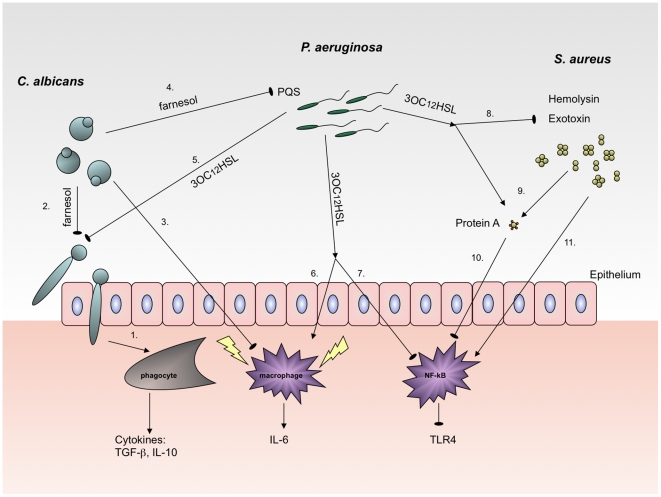
Effects of 3OC_12_HSL on *P. aeruginosa*, *S. aureus*, and *C. albicans* that are relevant to immune recognition. Lines ending in arrows indicate induction and lines ending in circles indicate inhibition of the indicated process. *C. albicans*, in the hyphal morphology, induces phagotycosis during inflammation (1). Virulent hyphae can be inhibited and transformed to yeast cells by farnesol, the QS molecule secreted by *C. albicans* (2). *Candida* yeast cells prevent macrophages induction (3) and render *C. albicans* invisible for the immune system. *C. albicans* coexisting with *P. aeruginosa* exerts a double-sided reaction; farnesol inhibits *Pseudomonas* QS production (4), whereas 3OC_12_HSL secreted by *P. aeruginosa* prevents *C. albicans* filamentation without changing the growth rate (5). 3OC_12_HSL sensed by the host induces a pro-inflammatory response by activation of macrophages (6), but it can also give an anti-inflammatory reaction by selectively diminishing the regulation of NF-κB signaling and attenuating TLR4-dependent innate immune responses (7). *P. aeruginosa* 3OC_12_HSL influences *S. aureus* by inhibiting growth and hemolysin and exotoxin production (8) and by inducing protein A expression (9), which prevents recognition of *S. aureus* by macrophages and neutrophils (10). *S. aureus* detected by the immune system trigger macrophages signaling pathways (11).

3OC_12_HSL is secreted in considerable amounts by *P. aeruginosa* during growth and is therefore readily detected by the host. Several host cell types, including macrophages and epithelial cells, have been shown to respond to synthetic 3OC_12_HSL, resulting in both a pro- and anti-inflammatory immune-modulatory response [5]. For example, detection of 3OC_12_HSL by corneal epithelial cells of the eye results in production of the macrophage attractant cytokine IL-6, creating a strong pro-inflammatory response (Figure 2) [6]. Although intercepting and deciphering microbial communication by epithelial and immune cells could be beneficial for the host, it is important to note that activation of the host immune system could also be beneficial for the pathogen, as over-stimulation of the inflammatory process results in extensive tissue damage. Moreover, 3OC_12_HSL selectively diminishes the regulation of NF-κB signaling and attenuates TLR4-dependent innate immune responses, which potentially promote infection persistence, particularly in cystic fibrosis patients [7]. The production of 3OC_12_HSL by *P. aeruginosa* also suppresses the activation of immune cells and induces apoptosis in macrophages, thereby compromising host immune defenses.

## Interspecies Sensing: Detection, Hiding, and Early Warning: *P. aeruginosa* – *S. aureus*

Similar to host immune cells, competing bacterial species have developed ways to detect *P. aeruginosa* through secreted 3OC_12_HSL. Specifically, the bacterial pathogen *S. aureus* has been shown to respond to the presence of *P. aeruginosa* [8]. An important staphylococcal surface protein, protein A, involved in *S. aureus* defense against the host immune system is up-regulated in response to 3OC_12_HSL (Figure 2). Binding of the Fc receptor on immunoglobulin G by protein A prevents recognition of *S. aureus* by macrophages and neutrophils. This could represent a mechanism by which *S. aureus* prepares itself for an attack of the host immune system.

3OC_12_HSL induces down-regulation of the *sarA* and *agr* genes and consequently, several virulence factors, such as hemolysin, exotoxin, and fibronectin-binding protein, and factors related to biofilm formation are down-regulated in *S. aureus* in response to *P. aeruginosa* presence. This response of *S. aureus* to 3OC_12_HSL is specific, as no response was observed for 3OC_4_HSL, nor for unsubstituted acylhomoserine lactones (AHLs), such as C_12_HSL [8]. Because there is also evidence for binding of 3OC_12_HSL to a specific receptor in *S. aureus*, it is conceivable that *S. aureus* sensing of *P. aeruginosa*, via detection of 3OC_12_HSL, could be interpreted as a sophisticated early warning for *S. aureus* of the presence of a competitor as well as an onset of the host immune response.

## Inter-Kingdom Sensing:* P. aeruginosa – C. albicans*

*C. albicans* is a dimorphic fungus able to switch morphology between the yeast and hyphal forms [9], a property crucial to its pathogenesis (Figure 2). *P. aeruginosa* adheres to *C. albicans* hyphae but not to the yeast morphology, making only the hyphal morphology susceptible to killing by *P. aeruginosa* [10], [11]. The bacterial factors involved in adhesion to hyphal cells such as the chitin-binding protein (CbpD) are under QS regulatory control (E. Ovchinnikova, B.P. Krom, H.C. van der Mei, and H.J. Busscher, unpublished data). *Pseudomonas* 3OC_12_HSL not only regulates the adhesion capabilities to *C. albicans* hyphae, but also modulates the *C. albicans* morphological switch by preventing the yeast-to-hypha transition [12], and *C. albicans* interprets 3OC_12_HSL as a warning signal.

## Interfering with Communication as Potential Therapeutic Strategies

In light of the importance of information exchange, it is not surprising that different systems have developed that interfere with the successful exchange of information. Several mechanisms have been described in the literature, ranging from enzymatic degradation of the signaling molecule to competitive inhibition of receptor binding.

### Enzymatic Interference

Many bacteria possess genes that encode lactonases or acylases [13]. These enzymes can deactivate 3OC_12_HSL and in turn interfere with communication. In fungi, AHL hydrolyzing activity has been observed in *Ascomycetes* and *Basidiomycetes* [14]. Although only C_6_HSL and 3OC_6_HSL have been shown to be hydrolyzed by these fungi, this example illustrates that fungi have developed systems to interfere with bacterial QS-mediated communication. Similarly, humans have also developed the ability to hydrolyze AHLs via a class of enzymes called paraoxonases (commonly referred to as PONs) [13]. In a *Drosophila* infection model it was shown that human PONs are protective against *P. aeruginosa* infections.

### Non-Enzymatic Interference

*C. albicans* secretes farnesol, a QS molecule similar in structure to 3OC_12_HSL, which at low cell density inhibits *Pseudomonas* quinolone signal (PQS) production [15] required for the expression of several virulence factors (Figure 2) [16]. At higher concentration, this molecule can suppress the effect of farnesol on PqsR activity [15]. Recently, it was reported that farnesol has a stimulating effect on PQS production in a *P. aeruginosa lasR* mutant, indicating that there is a specific target for that interaction [17]. *P. aeruginosa lasR*–deficient mutants arise frequently during chronic infection, likely due to selective advantages in growth, death, and lysis over wild-type cells [18], which suggests that 3OC_12_HSL might be dispensable for *P. aeruginosa* virulence. This phenomenon may also reflect the negative effects of the host response to 3OC_12_HSL on *P. aeruginosa* survival.

In summary, in addition to playing a pivotal role in virulence, the secreted *P. aeruginosa* QS molecule 3OC_12_HSL modulates the host immune response to *P. aeruginosa* infection and influences the virulence of other opportunistic pathogens. In conclusion, the identification of QS as a microbial strategy to control virulence by means of extracellular signal molecules has identified these molecules as potential drug targets for blocking pathogenicity.
